# Effects of a Benzimidazolium Salt on Colorectal Cancer Cells: Involvement of Apoptosis and Autophagy

**DOI:** 10.3390/molecules31203606

**Published:** 2026-10-10

**Authors:** Mustafa Cakir, Eda Acikgoz, Burak Kuzu, Sakine Akar, Halil Ozkol, Hamiyet Donmez-Altuntas, Senem Akkoc

**Affiliations:** 1 Department of Medical Biology, Faculty of Medicine, Van Yuzuncu Yıl University, Van 65100, Türkiye; 2 Department of Histology and Embryology, Faculty of Medicine, Van Yuzuncu Yıl University, Van 65100, Türkiye; 3 Department of Pharmaceutical Chemistry, Faculty of Pharmacy, Van Yuzuncu Yıl University, Van 65100, Türkiye; 4 Department of Medical Biology, Faculty of Medicine, Erciyes University, Kayseri 38039, Türkiye; donmezh@erciyes.edu.tr; 5 Department of Basic Pharmaceutical Sciences, Faculty of Pharmacy, Suleyman Demirel University, Isparta 32260, Türkiye; 6 Faculty of Engineering and Natural Sciences, Bahçeşehir University, Istanbul 34353, Türkiye

**Keywords:** colon cancer, benzimidazole, apoptosis, autophagy

## Abstract

Colorectal cancer (CRC) remains a leading cause of cancer-related mortality worldwide, necessitating the identification of novel small molecules capable of targeting multiple survival pathways. In this study, the antiproliferative potential of dds benzimidazole-palladium (Pd) complexes was evaluated across breast cancer (4T1, MDA-MB-231) and CRC (DLD-1, HT-29) cell lines using MTT assays. Following initial screening, CRC models were prioritized for mechanistic investigation due to the complexes’ superior cytotoxic profile, particularly that of the lead candidate **3a**, which exhibited significant dose-dependent efficacy. Mechanistic analyses, employing immunofluorescence staining for LC3 and mTOR alongside Annexin V/PI flow cytometry, revealed that compound **3a** triggers a potent dual response characterized by concomitant autophagy and apoptosis. Furthermore, functional assays indicated that **3a** significantly impairs the metastatic hallmarks of CRC cells by inhibiting migration, cell adhesion, and colony formation. Gene expression profiling further elucidated that the anticancer efficacy of **3a** is mediated by suppression of the AKT/mTOR/STAT3 signaling axis, thereby triggering an autophagic response that destabilizes cell adhesion and accelerates apoptosis. These molecular findings were corroborated by in silico molecular docking analyses, which demonstrated favorable binding affinities between compound **3a** and key regulatory proteins, specifically BCL-2 and Caspase-3. Collectively, these results identify compound **3a** as a promising multi-targeted anticancer agent that disrupts critical survival and metastatic pathways in CRC, warranting further preclinical and mechanistic exploration for therapeutic development.

## 1. Introduction

Colorectal cancer (CRC), the third most common cancer worldwide, is a global health problem resulting in approximately 1.9 million new cases and 930,000 deaths according to 2020 data [1]. Current demographic projections predict that this burden will reach 3.2 million new cases by 2040 [1]. Current therapeutic strategies largely focus on active cell proliferation; however, limitations such as acquired chemoresistance and off-target systemic toxicities necessitate the design of novel synthetic compounds with enhanced specificity and therapeutic efficacy [2].

Benzimidazole, a key pharmacophore with an aromatic heterocyclic core, is widely used in drug discovery [3]. This framework has served as the basis for numerous synthetic derivatives exhibiting a variety of biological activities, including antibacterial, antiviral, antifungal, and antiproliferative effects [4]. Benzimidazolium salts, in particular, have attracted attention as target-based chemical frameworks for cancer treatment and have been studied in various cancer cell lines [5,6]. Benzimidazolium salts exhibit potent anticancer effects by inhibiting cancer cell proliferation, inducing apoptosis, and modulating critical cellular signaling pathways [7,8]. These molecules possess multifaceted mechanisms for cell cycle arrest through DNA binding, disruption of microtubule dynamics, and specific enzyme inhibition [9]. In addition to these comprehensive effects of benzimidazolium salts, the inhibitory roles of benzimidazolium salts, particularly on the STAT3 signaling pathway, have been demonstrated [10].

Autophagy is a bidirectional mechanism in CRC that plays both suppressive and supportive roles in tumor development by regulating stress adaptation [11]. In CRC, autophagy is a central survival mechanism tightly controlled by the interaction between the STAT3 and mTOR signaling pathways. The fact that STAT3 inhibition triggers autophagy by suppressing mTOR activity makes this axis a critical molecular target for tumor progression and treatment response [12]. The lethal autophagy mechanism, as it is called, can transform this process into a death pathway when cellular stress exceeds a certain threshold [13]. This phenomenon triggers pro-apoptotic signals from proteins such as Beclin-1 and Atg5, cleaved by caspases, leading the cell into an irreversible process of destruction. Consequently, the synergistic interaction between this initially protective mechanism and apoptosis presents a strategic therapeutic target that overcomes the chemoresistance shields of cancer cells and drives them into programmed cell death [14].

Further molecular and mechanistic studies supporting the anticancer effects of benzimidazolium salts are of great importance in determining the place of these compounds in the rational drug development process. This work begins with benzimidazoline salts synthesized by Akkoç, which were initially evaluated for efficacy using MTT-based cytotoxicity assays [15]. Our preliminary experimental observations revealed that these compounds not only reduce cell viability but also cause significant changes in cell morphology. In particular, the formation of autophagosome-like vacuolar structures and a significant decrease in cell adhesive properties after drug application were observed. This series, exhibiting low IC_50_ values and morphologically diverse effects, suggests that mechanisms beyond classical apoptotic cell death may be at play. Therefore, a more detailed evaluation of these compounds across different cancer cell types and elucidation of their mechanisms of action via apoptosis- and autophagy-related molecular pathways constitute the main objective of the present study.

## 2. Materials and Methods

### 2.1. Synthesis of Benzimidazole-Based Compounds

**Scheme 1:**
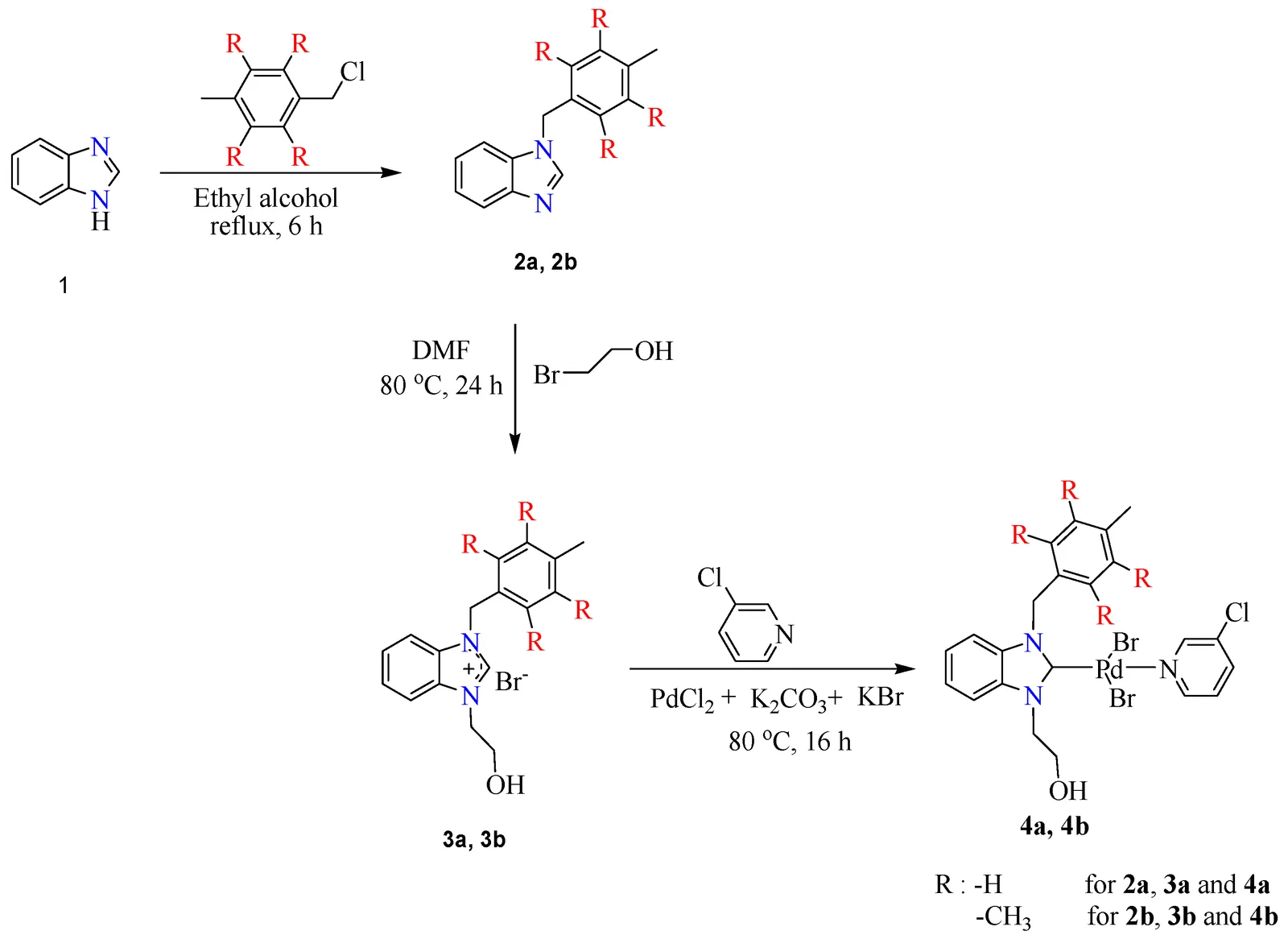
Synthesis of compounds **2a**–**b**, **3a**–**b**, and **4a**–**b**.

*o*-Phenylenediamine, 4-methylbenzyl chloride, PdCl_2_, and 3-chloropyridine used in this study were purchased from Merck, while 2,3,4,5,6-pentamethylbenzyl chloride and 2-bromoethanol were obtained from Sigma-Aldrich. All solvents and other chemical reagents were commercially obtained from Merck, Sigma-Aldrich, and Scharlau and used as received without further purification. All synthesis procedures were performed using a Schlenk line under an inert argon atmosphere, where required, to minimize exposure to atmospheric moisture and oxygen during the reactions and subsequent handling of the reaction mixtures. The 2-hydroxyethyl-substituted benzimidazolium salts and the corresponding palladium complexes evaluated in this study were synthesized by our research group as previously reported [15] (Scheme 1). In the final step, 3-chloropyridine served as both the reactant and the solvent for the conversion of **3a** and **3b** to the corresponding palladium complexes **4a** and **4b** and was therefore used in excess to facilitate the reaction (Scheme 1). After completion of the reaction, the products were purified by column chromatography, and the excess 3-chloropyridine was subsequently removed under vacuum. Detailed synthetic procedures, structural characterization (^1^H NMR, ^13^C NMR, FT-IR, elemental analysis), physical properties, and fundamental chemical data of these compounds have been reported previously [15]. Compounds **3a**, **3b**, **4a**, and **4b** were obtained in yields of 58%, 57%, 49%, and 21%, respectively.

### 2.2. Cell Culture

The human breast cancer cell line MDA-MB-231 (HTB-26), the mouse breast cancer cell line 4T1 (CRL-2539), and the human CRC cell lines DLD-1 (CCL-221) and HT-29 (HTB-38) were obtained from the American Type Culture Collection (ATCC). MDA-MB-231, 4T1, DLD-1 and HT-29 cells were cultured in a single layer in RPMI 1640 or Dulbecco’s Modified Eagle Medium (DMEM, Gibco) supplemented with 10% fetal bovine serum (FBS, Gibco), 4.5 g/L L-glutamine, 1 mM sodium pyruvate, 100 U/mL penicillin-streptomycin and 1% amphotericin B. Cultures were maintained at 37 °C in a humidified incubator containing 5% CO_2_.

### 2.3. MTT Cell Viability Test

The cytotoxic effect of compounds **3a**, **3b**, **4a** and **4b** on MDA-MB-231, 4T1, DLL-1, and HT-29 cells was evaluated using the MTT [3-(4,5-dimethylthiazol-2-yl)-2,5-dipheny ltetrazolium bromide] test. Cells were seeded into 96-well plates at a cell density of 6 × 10^3^ per well and incubated overnight. They were then treated with various concentrations of the compound (1–160 µM) for 48 h. After treatment, 10 µL of MTT (5 mg/mL) solution was added to each well, and the plate was incubated at 37 °C for 4 h. 50 µL of DMSO was added, and absorbance was measured at 570 nm using a microplate reader (Biobase, BK-EL-10C, Jinan, China). Cell viability was expressed as a percentage relative to an untreated control group, which was considered 100%.

### 2.4. Immunofluorescence (IF) Staining

HT-29 and DLD-1 colorectal cancer cells were seeded onto sterile glass coverslips in 6-well culture plates and subsequently treated with **3a** under the specified experimental conditions. Upon completion of the incubation period, cells were fixed in 4% paraformaldehyde for 15 min at room temperature and rinsed thoroughly with phosphate-buffered saline (PBS). Cellular permeabilization was achieved using Triton X-100 for 10 min at room temperature, followed by blocking of non-specific antibody binding with 5% bovine serum albumin (BSA). Immunostaining was performed by incubating the cells overnight at 4 °C in a humidified chamber with primary antibodies targeting LC3 (bs-8878R, Bioss Antibodies Inc., Woburn, MA, USA) and mTOR (bs-1992R, Bioss Antibodies Inc., Woburn, MA, USA), each diluted 1:200 in blocking buffer. After extensive washing with PBS, cells were incubated with a FITC-conjugated secondary antibody (#31583, Invitrogen/Thermo Fisher Scientific, Waltham, MA, USA) for 1 h at room temperature. Nuclear counterstaining and mounting were performed with a DAPI-containing mounting medium (sc-24941, Sigma, Dallas, TX, USA). Fluorescence images were acquired using an Olympus BX53 fluorescence microscope equipped with a DP74 camera system (Olympus, Tokyo, Japan). Quantitative image analysis was conducted using ImageJ software (version 2.16.0/1.54p). Mean fluorescence intensity values were determined from 30 cells per condition, selected from randomly chosen microscopic fields.

### 2.5. Annexin V Apoptotic Analysis

To evaluate the apoptotic effects of compound **3a**, DLD-1 and HT-29 cells were treated for 48 h and then stained with Annexin V-APC and Propidium Iodide (PI). After staining, they were analyzed by flow cytometry to distinguish between viable and apoptotic cell populations. Apoptotic percentages were measured for each group and compared with controls.

### 2.6. Cell Migration Experiment

A wound healing experiment was performed to evaluate the effects of compound **3a** on cell migration. Details of the method are given in our previous study [16]. Briefly, HT-29 CRC cells were seeded into 6-well plates at a density of 10 × 10^5^ cells per well. After the cells reached approximately 70–80% density, at the end of 48 h of incubation, a wound was created on the culture surface using a sterile 100 µL pipette tip. Wound widths were measured at 0 and 48 h, and percentages were calculated using ImageJ.

### 2.7. Cell Adhesion Experiment

HT-29 CRC cells were treated with compound **3a** for 24 h. Control group cells received no treatment. Then, the cell adhesion experiment was performed as described in the literature [17]. Microplate wells were coated with 1% Poly-D-lysine and blocked with heat-denatured bovine serum albumin (BSA) to prevent non-specific binding. After fixing the cells with methanol, they were stained with 0.1% crystal violet, and the amount of bound cells was visualized by removing excess dye. In the final step, the dye bound to the cells was dissolved in 10% acetic acid and analyzed spectrophotometrically at 570 nm using a microplate reader.

### 2.8. Gene Expression Analysis

HT-29 cells were incubated with compound **3a** at an IC_50_ (3.7 µM) concentration for 48 h, followed by total RNA isolation using Trizol reagent (Invitrogen, Waltham, MA, USA) according to the manufacturer’s instructions. cDNA synthesis was performed from the isolated RNA samples using reverse transcriptase enzyme; then, the expression levels of apoptosis-related (BCL-2, BAX, and Caspase-3), autophagy-related genes (Mtor and BECN1), adhesion-related (VIM and ITGβ1), AKT, and Stat3 genes were analyzed using quantitative real-time PCR (qPCR). Gene expression data were normalized relative to the beta-actin gene used as an internal control, and relative mRNA changes were calculated using the 2^−∆∆Ct^ method. For statistical comparisons, the control group expression level was fixed at 1, and fold changes in target gene expression upon **3a** application were determined relative to this reference value.

### 2.9. Molecular Docking Analysis

Molecular docking studies were conducted using AutoDock 4.2 software to identify the interactions of compound **3a** against 3D structures of BAX, BCL-2, and Caspase-3. The crystal structures of BAX (PDB ID: 4S0O), BCL-2 (PDB ID: 4MAN), and Caspase-3 (PDB ID: 5I9B) were obtained from the RCSB Protein Data Bank (www.rcsb.org). The molecular structure of compound **3a** was drawn using GaussView 5.0 and then optimized using the DFT method with the Gaussian 03 package at the B3LYP/6–31G level. Docking processes started with removing the unwanted solvent, water, and ligands from the BAX, BCL-2, and Caspase-3 crystal structures. In docking studies, the coordinate centers were determined as BAX: x: 14.642; y: −0.204; z: 15.731; BCL-2: x: −12.621; y: 8.675; z: 7.516; and Caspase-3: x: −3.156; y: −13.42; z: −18.754 [18]. Then, using a 40 × 40 × 40-point grid centered on the predicted locations and a grid point spacing of 0.375 Å, the lowest-energy conformations were selected for further study. Water molecules were removed with AutoDock tools, and subsequently, polar hydrogen atoms, Gasteiger partial charges, and Kollman charges were added to the targets. Additionally, the rotatable bonds of the compounds were adjusted. The target regions of compound **3a** were docked under validated conditions using AutoDock 4.2. To validate the docking process for the specified target, the native ligand was redocked and overlapped with the experimentally determined crystal structure. The Lamarckian genetic algorithm (10 runs) approach was applied, with a population size of 300 in both simulations. The interactions of the crystal structure of BAX, BCL-2, and Caspase-3 with the compound **3a** were analyzed using the Discovery Studio Client 4.1 program.

### 2.10. Statistical Analysis

Statistical analyses were performed using GraphPad Prism 8.1.0 software. Student’s t-test was applied for pairs of samples. For data with more than two samples, one-way ANOVA followed by Tukey’s multiple comparison test was applied; IC_50_ values and confidence intervals were obtained from nonlinear regression analyses. A *p*-value < 0.05 was considered statistically significant.

## 3. Results

### 3.1. 2-Hydroxyethyl Substituted Benzimidazolium Salts Demonstrate Significant Anti-Cancer Efficacy

**Figure 1:**
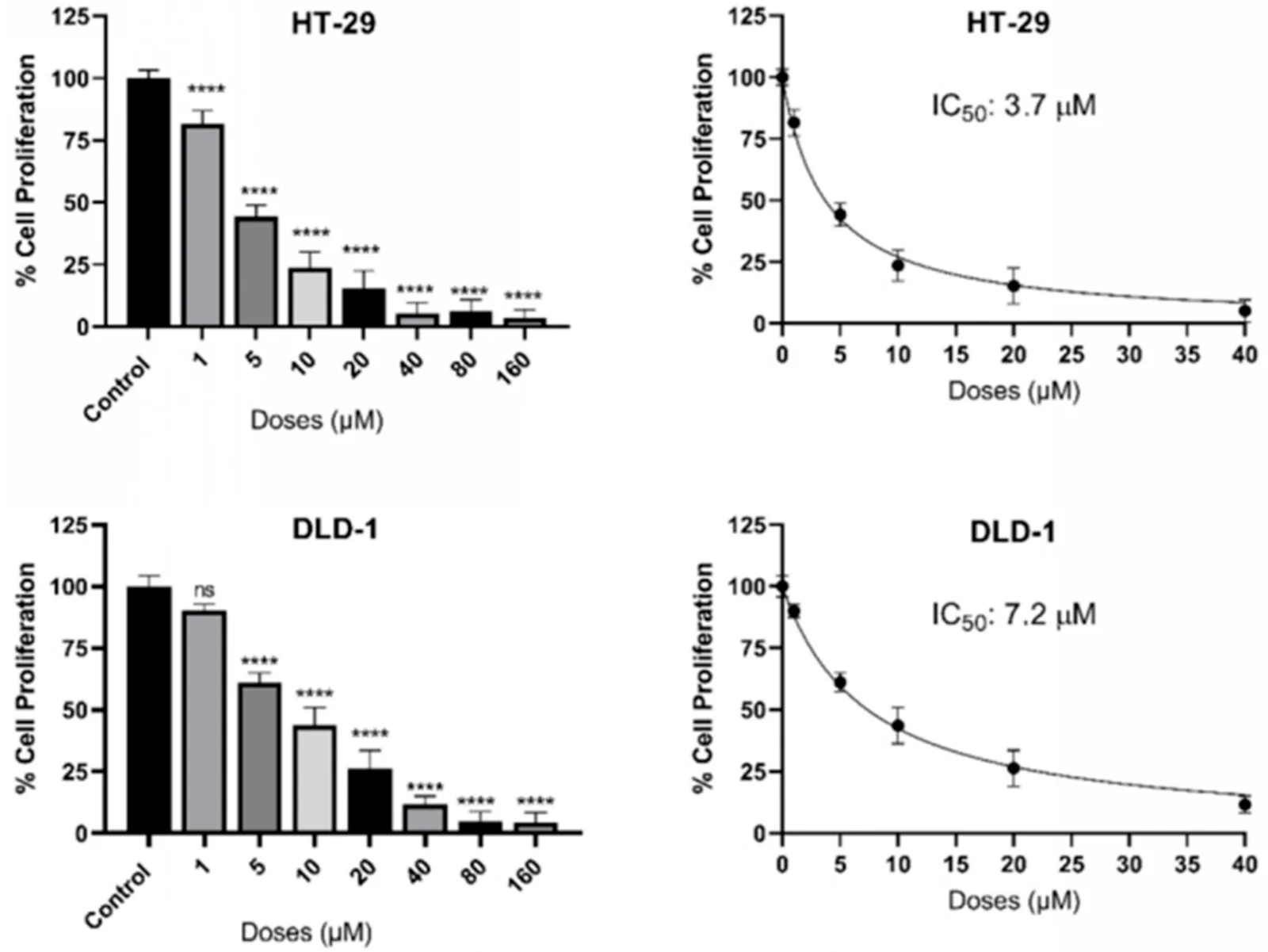
Cytotoxic profile and dose–response curves of compound **3a** in colorectal cancer cells. The bar charts and non-linear regression curves illustrate a significant, dose-dependent inhibition of cell proliferation in both HT-29 and DLD-1 cell lines after treatment. While compound **3a** exhibited high potency across both lines, HT-29 cells showed greater sensitivity with an IC_50_ of 3.7 µM, compared to 7.2 µM in DLD-1 cells. Statistically significant differences compared to the control group are indicated as ns: non-significant and **** *p* < 0.0001.

In the previous study, the cytotoxicity of compounds **3a**–**b** and **4a**–**b** was tested in DLD-1 and MDA-MB-231 cells [15]. In this study, cytotoxicity tests were performed in four different cell lines, including 4T1 mouse breast cancer cells and HT-29 human CRC cells. In all four cell lines, the lowest IC_50_ values were observed in compound **3a** (Table 1). The cytotoxic potential of compound **3a** was evaluated against HT-29 and DLD-1 colorectal cancer cell lines using increasing concentrations (1–160 µM). Our results demonstrated that **3a** significantly suppressed cell proliferation in a dose-dependent manner in both models (Figure 1). Notably, HT-29 cells exhibited a more sensitive profile with an IC_50_ value of 3.7 µM, while DLD-1 cells showed a slightly higher resistance with an IC_50_ value of 7.2 µM (Figure 1). Remarkably, the antiproliferative efficacy of compound **3a** in HT-29 cells was comparable to that of cisplatin, a widely used reference chemotherapeutic agent. These findings indicate that compound **3a** possesses strong antiproliferative activity at low micromolar concentrations, establishing a robust foundation for further mechanistic investigations into its pro-apoptotic and autophagic effects.

### 3.2. Coordinated Regulation of LC3 and mTOR by ***3a*** Induces Cytoprotective Autophagy in CRC Cells

**Figure 2:**
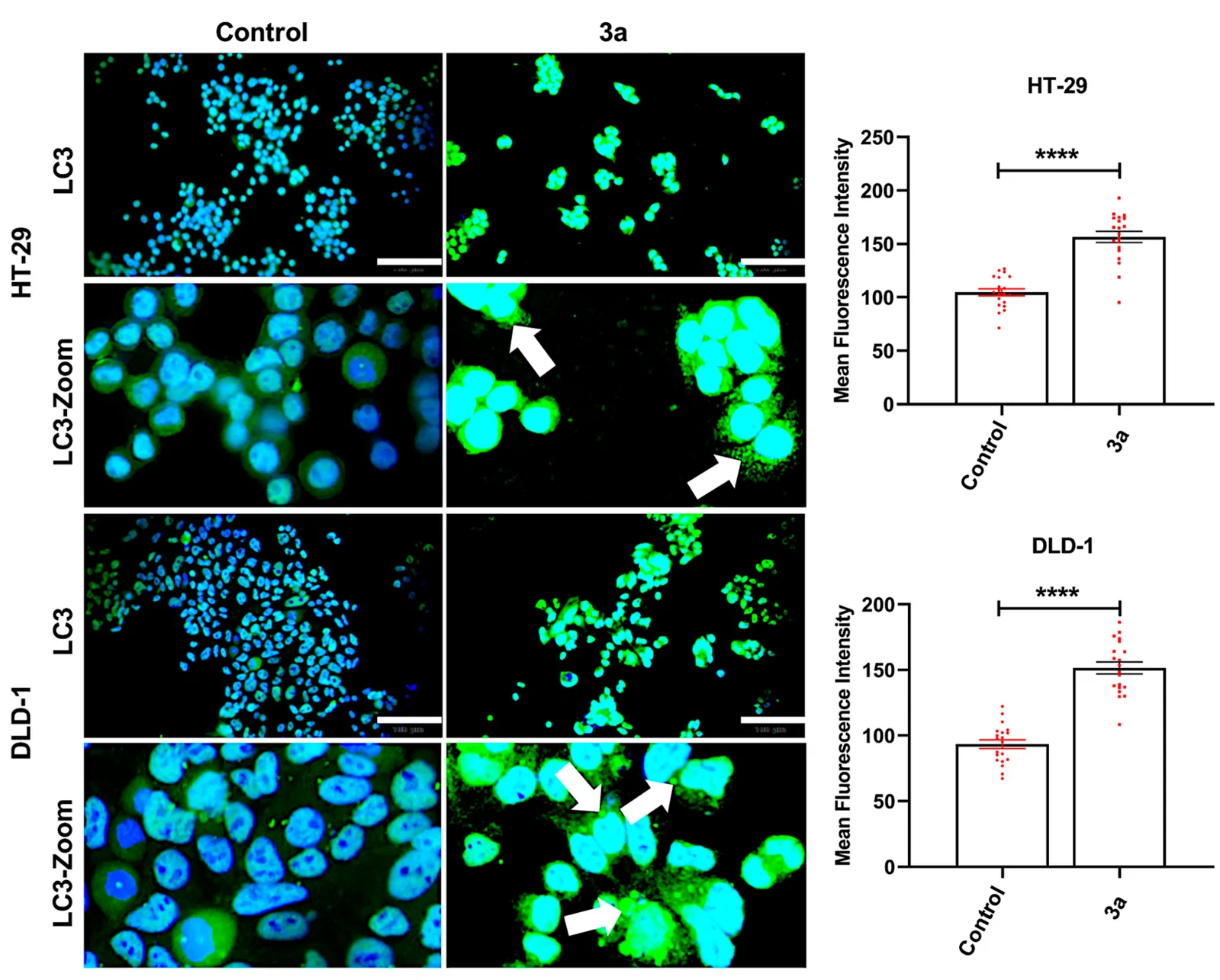
**3a** treatment enhances LC3 expression and induces punctate LC3 accumulation in colorectal cancer cells. Representative immunofluorescence images of LC3 staining in HT-29 and DLD-1 colorectal cancer cells under control conditions and following **3a** treatment. Low-magnification images and corresponding high-magnification (zoom) views illustrate LC3 localization patterns. Quantitative fluorescence analysis revealed a significant increase in LC3 mean fluorescence intensity in both HT-29 and DLD-1 cells upon **3a** exposure compared to controls (**** *p* < 0.0001). Notably, **3a**-treated cells exhibited prominent punctate LC3-positive structures within the cytoplasm (indicated by arrows), in contrast to the predominantly diffuse LC3 distribution observed in control cells. Nuclei were counterstained with DAPI. Scale bar: 100 µm.

Given the context-dependent role of autophagy in colorectal cancer progression and therapy response, immunofluorescence analyses of LC3 and mTOR were performed to evaluate whether **3a** treatment modulates key regulators of autophagy-associated signaling. In HT-29 cells, **3a** exposure resulted in a pronounced increase in LC3 fluorescence intensity compared to control cells, an effect that was statistically significant (*p* < 0.0001; Figure 2). A similar enhancement of LC3 signal intensity was consistently observed in DLD-1 cells following **3a** treatment, indicating a reproducible response across both colorectal cancer cell lines. Beyond quantitative changes, LC3 staining in **3a**-treated cells exhibited a marked alteration in subcellular distribution. Specifically, LC3 displayed a prominent punctate cytoplasmic pattern, characterized by dot-like aggregates dispersed throughout the cytoplasm, in contrast to the predominantly diffuse, low-intensity staining observed under control conditions (Figure 2).

**Figure 3:**
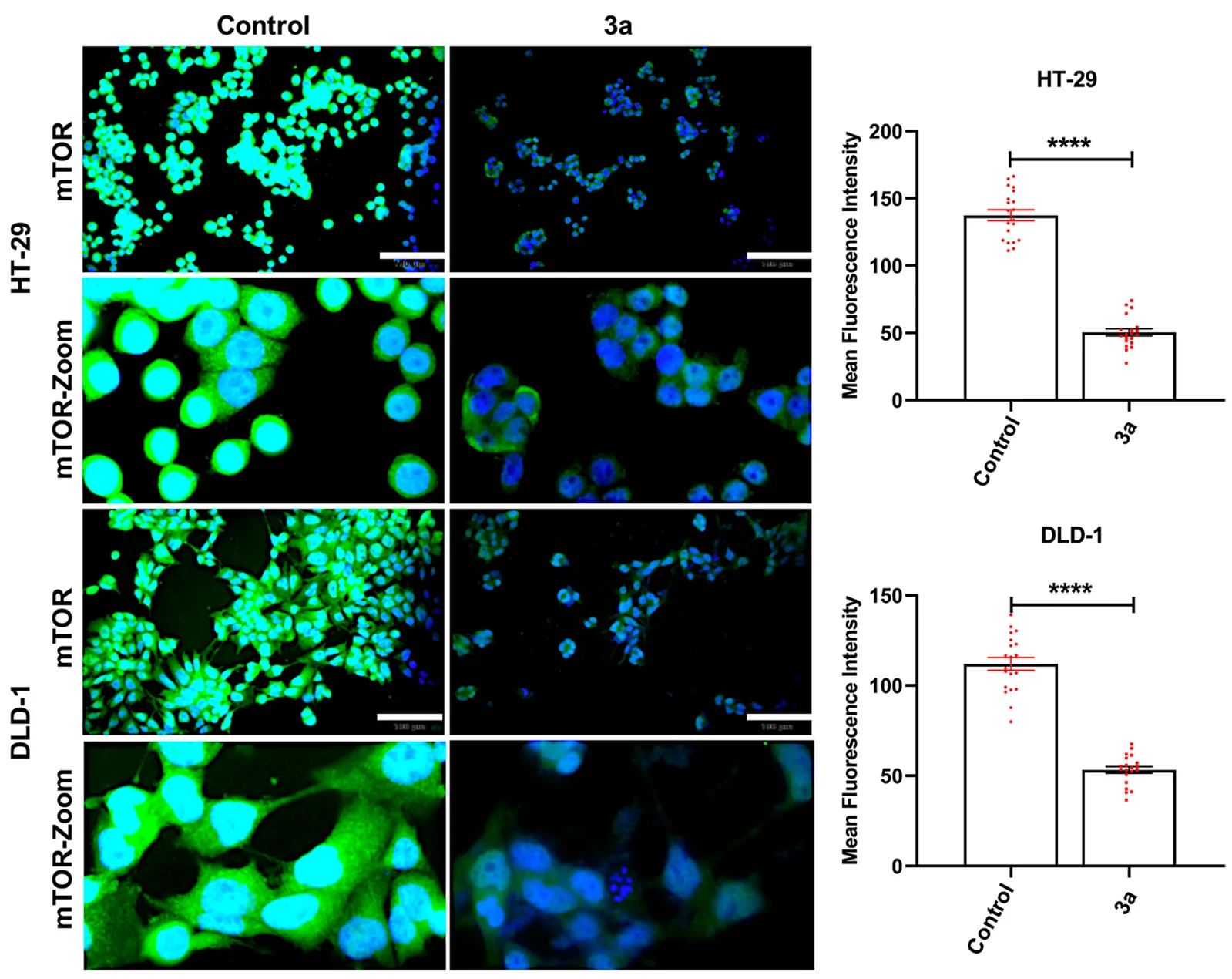
**3a** treatment reduces mTOR fluorescence intensity in colorectal cancer cells. Representative immunofluorescence images depicting mTOR expression in HT-29 and DLD-1 cells under control conditions and following **3a** treatment, shown at low magnification and corresponding zoomed-in views. Quantitative analysis demonstrated a significant reduction in mTOR mean fluorescence intensity in both colorectal cancer cell lines upon treatment with **3a**, compared with control cells (****, *p* < 0.0001). The decrease in mTOR signal intensity was consistently observed across independent experiments. Nuclei were counterstained with DAPI. Scale bar: 100 µm.

In parallel, immunofluorescence analysis of mTOR revealed an opposite response to **3a** treatment. In both HT-29 and DLD-1 cells, mTOR fluorescence intensity was significantly reduced following **3a** exposure when compared to control cells (*p* < 0.0001; Figure 3). This decrease in mTOR signal was consistently detected across independent experiments, suggesting a robust treatment-associated modulation of mTOR-related signaling. Taken together, the observed increase in punctate LC3 accumulation accompanied by a concurrent reduction in mTOR expression indicates a coordinated cellular response to **3a** treatment in colorectal cancer cells.

### 3.3. Treatment of CRC Cells with ***3a*** Suppressed Migration, Colonization, and Adhesion Properties

**Figure 4:**
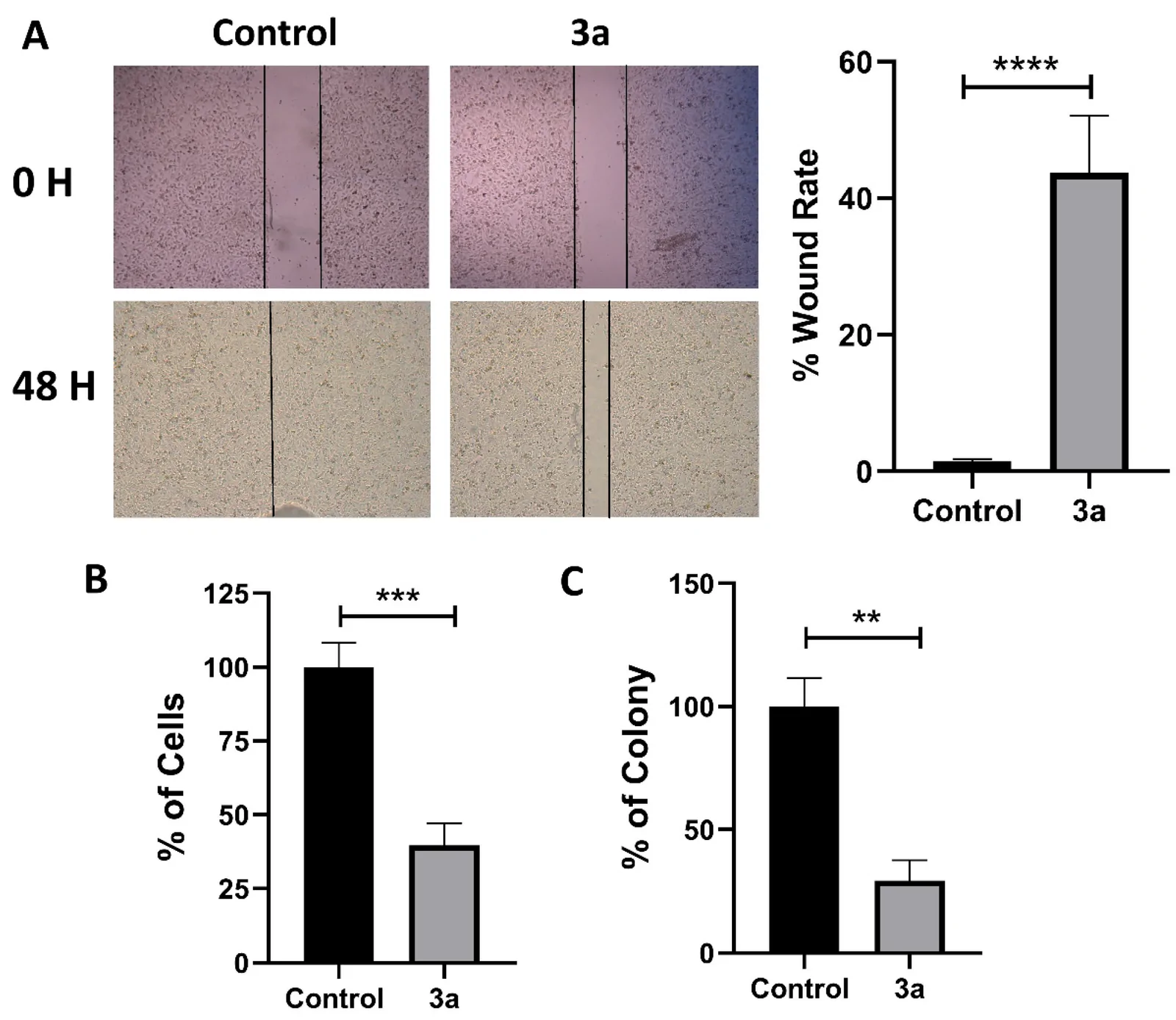
Treatment of CRC cells with **3a** decreased migratory, colonization, and adhesion characteristics. (**A**) Comparative analysis of the inhibitory effects of compound **3a** on the cell migration ability of HT-29 cells revealed that wound closure was not completed in the treatment group compared to the control group. This finding supports the strong potential anti-metastatic effect of **3a**. In the study, wound widths were analyzed using Image J. (**B**) A statistically significant decrease in the adhesion rate was observed between the control and treatment groups of HT-29 CRC cells treated with compound **3a**. (**C**) It is noteworthy that the treatment group showed a significant decrease in colony count in HT-29 cells compared to the control group. Data are presented as mean ± SD of three independent experiments. Statistically significant differences compared to the control group are indicated as ** *p* < 0.01, *** *p* < 0.001, and **** *p* < 0.0001.

To evaluate the effect of compound **3a** on the metastatic and tumor-forming properties of colorectal cancer cells, we performed a series of functional assays assessing migration, adhesion, and colony formation. The wound healing test showed that control HT-29 cells almost completely shut down within 48 h, while treatment with **3a** significantly inhibited lateral migration, leaving a significant gap and disrupting the cellular metastasis mechanism (Figure 4A). This anti-metastatic potential was further supported by a marked decrease in the percentage of adhering cells, suggesting that **3a** destabilizes cell–matrix interactions essential for survival and spread (Figure 4B). Furthermore, the colony formation test showed that **3a** significantly suppressed the ability of single cancer cells to proliferate into independent colonies, thus severely impairing the long-term tumor-forming and survival potential of the HT-29 cell line (Figure 4C). Collectively, these findings demonstrate that compound **3a** effectively attenuates the aggressive phenotype of CRC by inhibiting key processes such as colonization, adhesion, and migration.

### 3.4. Interference with the AKT/mTOR/STAT3 Signaling Axis Using a Benzimidazole Derivative Compound ***3a*** Restricts the Metastatic and Proliferative Potential of Human CRC Cells

**Figure 5:**
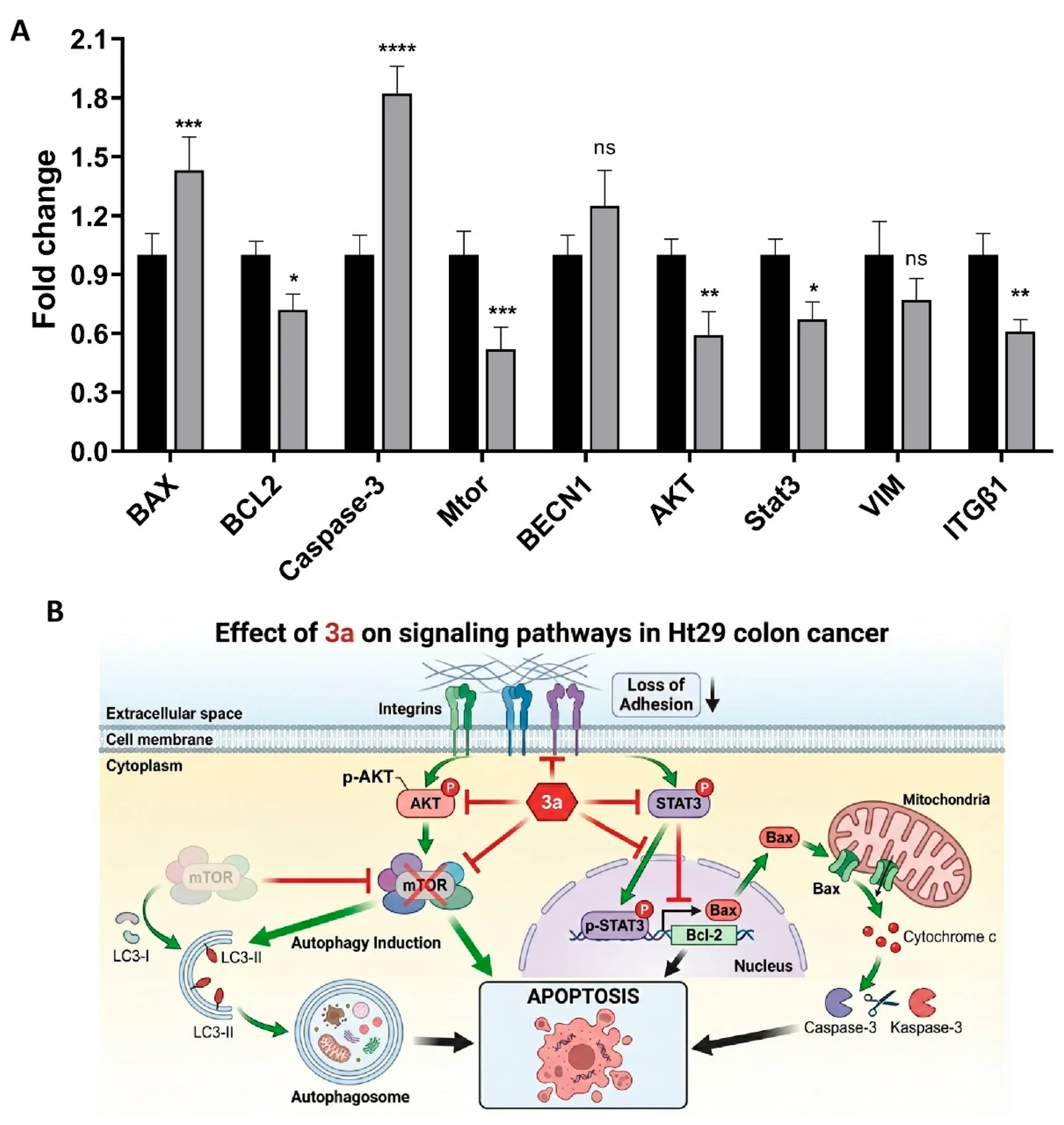
Gene expression profile of HT-29 cells treated with compound **3a**. (**A**) Black bars represent the control group, while gray bars indicate the compound **3a**-treated group. mRNA expression levels of key genes involved in apoptosis (BAX, BCL2, Caspase-3), autophagy (mtor, BECN1), survival signaling (AKT, Stat3), and adhesion (VIM, ITGβ1) were analyzed by RT-qPCR after 48 h of treatment with compound **3a**. GAPDH was used as the housekeeping gene. Fold-change values are shown with the control group fixed at 1. Significant downregulations were observed in the AKT/mTOR/STAT3 axis and adhesion markers, while pro-apoptotic markers were significantly upregulated. Statistically significant differences compared to the control group are indicated as ns; non significant, * *p* < 0.05, ** *p* < 0.01, *** *p* < 0.001, and **** *p* < 0.0001. (**B**) Schematic representation of altered gene expression in HT-29. Drawn by artificial intelligence based on information from the literature, then revised and checked before being added. Green arrows indicate activation/induction pathways, red T-shaped bars represent inhibition/suppression events, and black arrows indicate cell fate/downstream cellular processes.

Gene expression analyses performed in HT-29 cells confirmed that the molecular mechanism underlying the antitumor effect of molecule **3a** is directly related to the induction of apoptosis and the suppression of survival pathways (Figure 5A,B). Examination of RT-qPCR data revealed a significant increase in pro-apoptotic BAX and the key executive caspase, Caspase-3, mRNA levels, accompanied by a significant decrease in anti-apoptotic BCL2 expression, indicating that the compound strongly activates the mitochondrial apoptosis pathway (Figure 5A). At the cellular signaling level, the simultaneous decrease in the expression of AKT, Stat3, and mTOR genes, which have high oncogenic potential, demonstrates that **3a** centrally targets the proliferation and survival networks of cancer cells. Furthermore, the coexistence of the increased trend in the autophagy marker BECN1 levels and mTOR inhibition suggests that a cellular stress response is triggered; Downregulation of the ITGβ1 and VIM genes, associated with metastasis and cell adhesion, demonstrates that the molecule limits tumor invasion and the cell’s capacity to adhere to the environment at the molecular level (Figure 5A).

### 3.5. Compound ***3a*** Triggers Apoptosis in CRC

**Figure 6:**
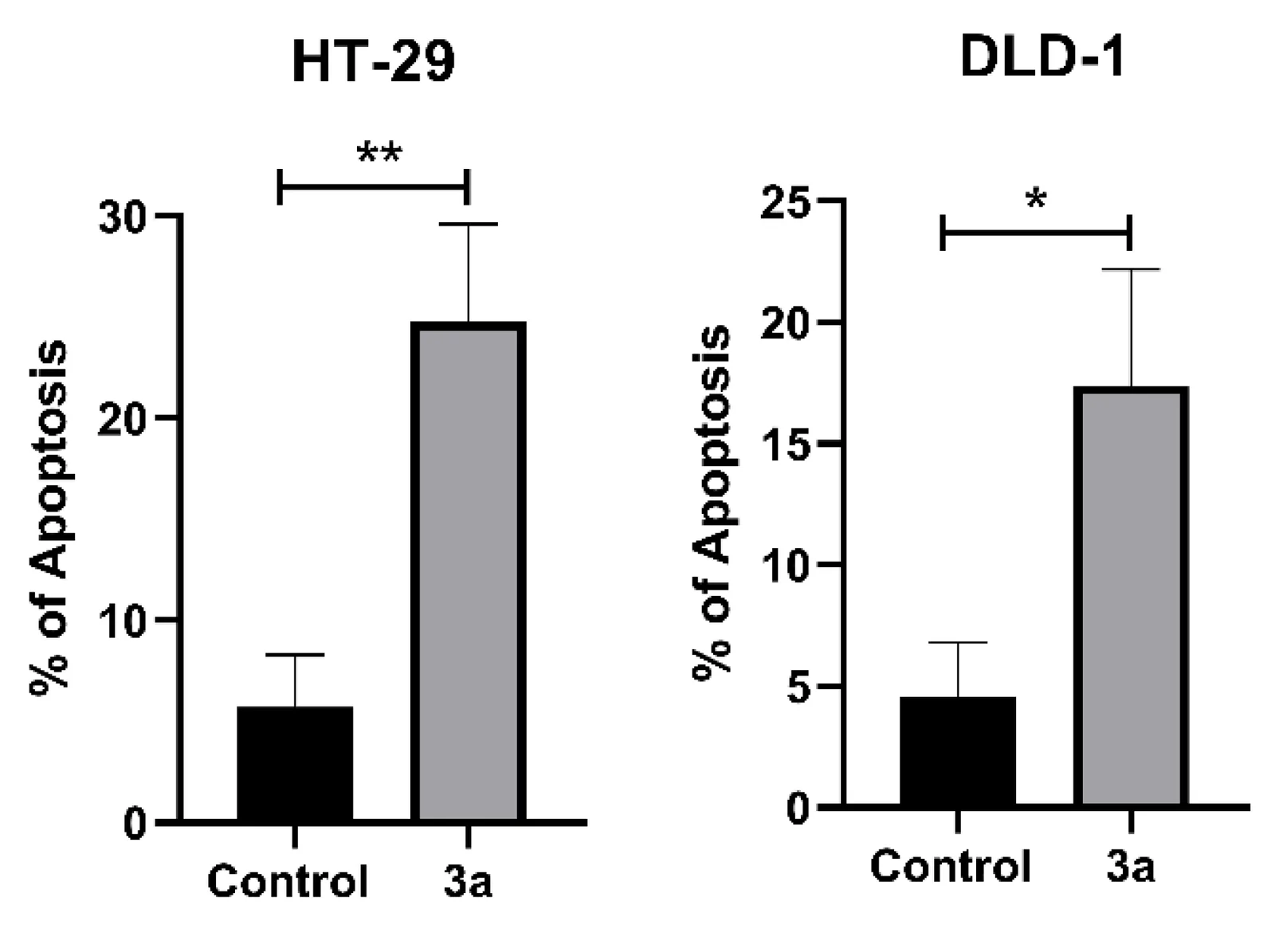
The effect of compound **3a** on apoptosis induction in colorectal cancer cells. HT-29 and DLD-1 colorectal cancer cells were treated with compound **3a** at the respective IC_50_ concentrations for 48 h. The percentage of apoptotic cells was determined by flow cytometry using Annexin V-FITC/PI double staining. Data are presented as mean ± SD of three independent experiments. Statistically significant differences compared to the control group are indicated as * *p* < 0.05 and ** *p* < 0.01.

Compound **3a** was shown to have a strong antiapoptotic effect, as evidenced by data from both qPCR and molecular docking. Annexin V-FITC/PI flow cytometry was used to assess, at the protein level, whether these cancer growth-inhibitory effects were mediated by programmed cell death. 48 h of treatment at IC_50_ concentration resulted in a significant shift from a viable cell population to an apoptotic population in both colorectal cancer cell lines (Figure 6). This confirmed that **3a** leads to activation of apoptosis, consistent with the results obtained throughout the study.

### 3.6. Molecular Docking Studies

Treatment of HT-29 cells with compound **3a** resulted in significant alterations in the gene expression profile of key regulators involved in apoptosis, autophagy, survival signaling, and cell adhesion. Notably, the expression levels of the pro-apoptotic genes BAX and Caspase-3 were markedly increased, whereas the anti-apoptotic gene BCL2 was significantly downregulated, indicating activation of apoptotic pathways. Given the critical roles of BAX in mitochondrial outer membrane permeabilization, BCL2 in inhibiting apoptosis, and Caspase-3 as an executioner caspase, these proteins were selected as primary targets for molecular docking analyses to further elucidate the molecular mechanisms underlying compound **3a**.

**Figure 7:**
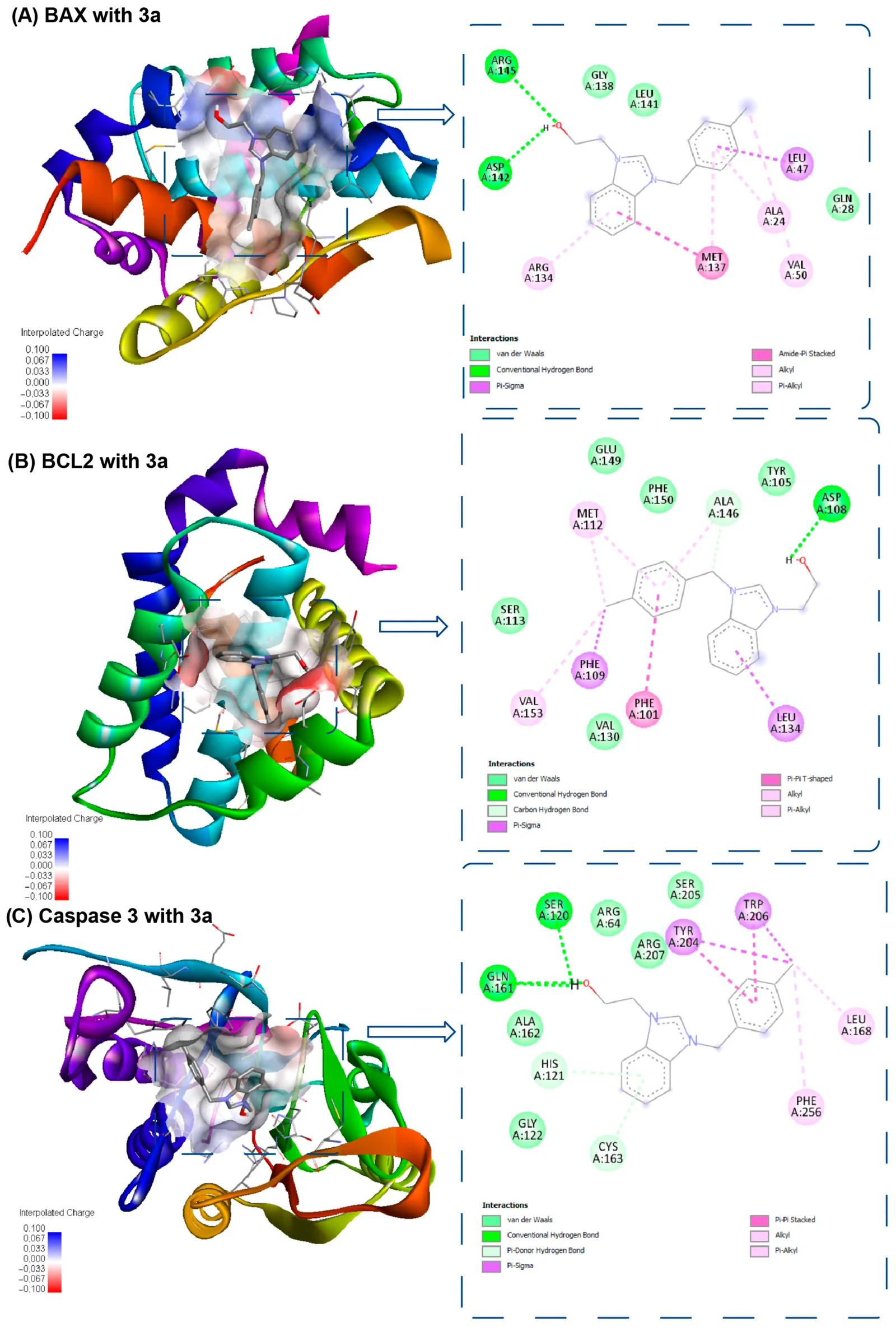
Molecular docking representations of compound **3a** with key apoptotic targets: (**A**) BAX, (**B**) BCL2, and (**C**) Caspase-3.

The docking results (Table 2) and visual representations of ligand–protein interactions (Figure 7) demonstrate that compound **3a** forms stable complexes with all three target proteins. The interaction with BAX exhibited a binding energy of −4.89 kcal/mol and an RMSD value of 0.14 A°, involving residues Ala24, Leu47, Val50, Arg134, Met137, Asp142, and Arg145 (Figure 7A). Although the binding affinity was comparatively lower, the very low RMSD value suggests a stable binding conformation. In contrast, docking with BCL2 yielded a binding energy of −6.06 kcal/mol and an RMSD of 0.59 A°, with key interactions involving Asp108, Phe101, Phe109, Met112, Ala146, and Val153 (Figure 7B). The strongest binding affinity was observed for Caspase-3 (−6.29 kcal/mol; RMSD: 0.39 A°), where compound **3a** interacted with residues Ser120, His121, Gln161, Cys163, Leu168, Tyr204, and Trp206 (Figure 7C). Notably, interaction with the catalytic residue Cys163 suggests a potential structural basis for modulation of Caspase-3 activity.

Overall, the higher binding affinities observed for BCL2 and Caspase-3 are consistent with the downregulation of BCL2 and upregulation of Caspase-3 at the gene expression level. Although a relatively weaker binding was detected for BAX, the formation of a stable complex indicates a possible contributory role in the apoptotic process. Collectively, these findings suggest that compound **3a** may exert its pro-apoptotic effects through multi-target modulation, particularly by influencing both mitochondrial pathways and caspase-mediated execution mechanisms.

## 4. Discussion

CRC has become a global health crisis and presents a difficult barrier to overcome in current treatment protocols, particularly due to chemoresistance mechanisms developing in metastatic processes [19]. Despite the widespread application of conventional cytotoxic agents, the ability of cancer cells to evade apoptosis through genomic instability and disrupt DNA repair mechanisms significantly limits the clinical efficacy of existing regimens. To overcome these treatment bottlenecks and control resistant cell populations, the discovery of innovative synthetic compounds that simultaneously target cellular survival and adhesion dynamics is an indispensable priority for modern oncology research [19,20]. Benzimidazole salts stand out for their superior solubility and stability, which are critical for overcoming the bioavailability hurdles of neutral compounds. Their unique ionic profile enables more precise interaction with cellular targets, effectively dismantling oncogenic signaling to drive resistant cancer cells toward programmed death [5,21].

In our previous study, the antiproliferative activities of 2-hydroxyethyl substituted benzimidazolium salts, which are 1-(2-hydroxyethyl)-3-(4-methylbenzyl)-1*H*-benzo[*d*]imidazol-3-ium bromide (**3a**), 1-(2-hydroxyethyl)-3-(2,3,4,5,6-pentamethyl benzyl)-1*H*-benzo[*d*]imidazol-3-iumbromide (**3b**), and their palladium complexes, which are dibromo-[1-(2-hydroxyethyl)-3-(4-methyl benzyl)benzimidazol-2-ylidene]-N-(3-chloropyridine) palladium (II) complex (**4a**), dibromo-[1-(2-hydroxyethyl)-3-(2,3,4,5,6-pentamethylbenzyl)benzimidazol-2-ylidene]-N-(3-chloropyridine) palladium (II) complex (**4b**), were evaluated in four different cancer cell lines (HeLa, MDA-MB-231, HepG2, and DLD-1) using the MTT assay method.

The results obtained revealed that the organic benzimidazolium salts possess significantly higher cytotoxic potential compared to their metal complexes. Specifically, compound **3b** exhibited measurable cytotoxic activity with an IC_50_ value of 80.64 ± 1.25 µM in the HeLa cell line, while the palladium complexes (**4a** and **4b**) did not show any antiproliferative effect in the same cell line. Similarly, compounds **3a** and **3b** showed moderate cytotoxic activity in HepG2 cells, while the metal complexes were also found to be ineffective against this cell line [15].

The results obtained in MDA-MB-231 and DLD-1 cell lines more clearly demonstrated the potent and selective antiproliferative effects of organic compounds. In MDA-MB-231 breast cancer cells, compound **3b** in particular showed the highest cytotoxic activity with an IC_50_ value of 7.59 ± 0.68 µM and was found to be more effective than cisplatin, which was used as a positive control. In addition, compounds **3a** and **3b** were found to have a dose-dependent effect against breast cancer cells. In the DLD-1 CRC cell line, compound **3a** showed a remarkable antiproliferative effect with an IC_50_ value of 9.19 ± 1.51 µM, which was very close to the IC_50_ value of cisplatin. In contrast, compounds **4a** and **4b** did not show cytotoxic activity in either cell line. These results indicate that the 2-hydroxyethyl substituted benzimidazolium salts synthesized in our previous study [15] are promising anticancer candidates, particularly MDA-MB-231 and DLD-1 cancer cells, and provide a strong basis for further biological studies.

Benzimidazole derivative drugs have been proven to have a highly effective anticancer effect in many cancers, particularly colon cancer [5,6,7,22]. Our previous study showed that organic benzimidazolium salts (**3a** and **3b**) have a much higher cytotoxic potential compared to metal complexes (**4a** and **4b**) and exhibit antiproliferative activity comparable to cisplatin, particularly in DLD-1 and MDA-MB-231 cell lines [15]. In this study, the effects of these four compounds on cell proliferation were investigated in aggressive mouse breast cancer cells (4T1), aggressive human breast cancer cells (MDA-MB231), and aggressive HT-29 and DLD-1 cells. Similar to the previous study, **3a** was the most effective compound in colon cancer, while **3a** and **3b** showed similar levels of anticancer activity in breast cancer. **4a** and **4b** did not show any anticancer activity, as in the previous study. In this case, the properties of benzimidazole suggest that the 2-hydroxyethyl substituent interacts more effectively with biological macromolecules in its free form, but complexation with palladium significantly reduces its anticancer activity by masking or halting the molecule’s active pharmacological sites. Kundu and colleagues synthesized two 1-(3-chlorobenzyl)-2-methyl-1*H*-benzo[*d*]imidazole derivatives, and the cytotoxic activities of these compounds were evaluated in HEK-293T, A549, HepG2, and MCF-7 cell lines. According to the results obtained, these two benzimidazole-based compounds exhibited significant cytotoxic effects, particularly in the HepG2 cell line (IC_50_: 21.11 and 17.19 µM) and the MCF-7 cell line (IC_50_: 16.82 and 19.38 µM). Furthermore, it was reported that these compounds exhibited lower cytotoxicity against the healthy HEK-293T cell line compared to cancer cell lines, thereby demonstrating a certain selectivity index [23]. In the study conducted by Akkoç and colleagues, benzimidazolium salts and their PEPPSI-type palladium N-heterocyclic carbene (NHC)-based complexes were synthesized. The cytotoxic effects of all compounds obtained were evaluated against HEK-293T, MDA-MB-231, and DLD-1 cell lines. They found that benzimidazolium salts exhibited higher cytotoxic activity against cancer cell lines compared to the corresponding metal complexes. In contrast, they determined that the synthesized palladium-NHC metal complexes did not show any significant cytotoxic effect against any of the tested cell lines [24].

Autophagy functions as a protective mechanism in the early stages of cancer, preventing tumor formation by maintaining genomic stability, while in advanced stages, its over-induction becomes a strategic therapeutic weapon, triggering programmed cell death by creating a metabolic impasse in cancer cells [25]. LC3, a specific marker of autophagosome formation, is widely used in autophagy measurements [26]. One of the striking findings of this study is that **3a** significantly induces autophagy. Immunofluorescence analyses show an increase in LC3 signaling and suppression of mTOR activity, indicating that **3a** activates the autophagic flow. Autophagy can play both tumor-suppressing and tumor-promoting roles in CRC, depending on the context. Particularly in advanced cancers, autophagy can support cell survival under metabolic stress, while excessive or dysregulated autophagy can lead to cell death [27]. In this study, the observation of autophagy induction along with apoptosis suggests that **3a** drives cells not towards protective autophagy, but towards a process described as “lethal autophagy.” Numerous protein molecules involved in tumor pathogenesis have been found to influence tumor formation by regulating autophagy and apoptosis; most of these are via the autophagy protein LC3, beclin-1, the apoptosis protein family Bcl-2, BAX, or the interaction between the two [28]. Autophagy and apoptosis are in constant interaction via common molecular bridges such as the BH3 domain and p53 to maintain cellular homeostasis and make decisions about survival or death under stressful conditions [29]. Apoptosis analyses showed that compound **3a** caused significant apoptotic cell death in both HT-29 and DLD-1 cells. This finding, confirmed by Annexin V/PI assays, is also supported by gene expression results. The decrease in anti-apoptotic BCL-2 expression, accompanied by an increase in pro-apoptotic BAX and caspase-3 expression, suggests that **3a** activates the intrinsic apoptotic pathway. Given that apoptotic mechanisms are frequently suppressed in CRC, contributing to chemotherapy resistance, the ability of **3a** to restore apoptotic balance is of therapeutic importance. It is thought that compound **3a** causes cell apoptosis by triggering lethal autophagy.

The AKT/mTOR/STAT3 signaling pathways are key regulators of proliferation, survival, migration, and chemotherapy resistance in CRC cells [30]. Gene expression analyses revealed that compound **3a** significantly suppressed AKT, mTOR, and STAT3 expression. The literature reports that constitutive activation of STAT3 is strongly associated with CRC progression, poor prognosis, and metastasis. STAT3 can also suppress autophagy by activating the mTOR pathway [31]. Therefore, the inhibition of the STAT3 and mTOR axis by compound **3a** is mechanistically consistent with the activation of autophagy and the attenuation of cellular survival signals.

Functional analyses demonstrate that molecular findings directly reflect cellular behavior. In wound-healing, cell adhesion, and colony formation experiments, **3a** was found to significantly inhibit the migration capacity, surface adhesion ability, and clonal growth of HT-29 cells. These findings are consistent with the decrease in Vimentin (VIM) and integrin β1 (ITGB1) expression observed in gene expression analyses. Integrins and EMT-related proteins play a critical role in CRC metastasis, and suppression of these proteins significantly reduces the invasive potential of tumor cells [32,33].

Research into the anticancer potential of benzimidazolium salts is receiving increasing attention, but detailed mechanistic evaluations are often lacking for many of these molecules [8]. This study is important because it shows that **3a** not only suppresses proliferation but also reverses the autophagy-apoptosis balance against the tumor by targeting the AKT/mTOR/STAT3 axis. Especially in a heterogeneous and treatment-resistant tumor type like CRC, such multi-target agents hold promise for next-generation therapeutic strategies.

## 5. Conclusions

Although this study has some limitations, the findings clearly demonstrate that compound **3a** exhibits a multifaceted anticancer effect in colon cancer cells. While the study was limited to in vitro models, the simultaneous induction of autophagy and apoptosis by **3a**, and the association of these processes with the suppression of AKT/mTOR/STAT3 signaling pathways, provides strong clues about the compound’s mechanism of action. Molecular docking analyses indicate that compound **3a** may induce apoptosis through multi-target interactions, based on favorable binding affinities with BAX (−4.89 kcal/mol), BCL2 (−6.06 kcal/mol), and Caspase-3 (−6.29 kcal/mol), providing a structural basis for its pro-apoptotic potential. Furthermore, the effective inhibition of migration, colony formation, and cell adhesion suggests that **3a** can also target cellular characteristics associated with metastasis.

A benzimidazole derivative compound **3a** emerges as a promising anticancer candidate that suppresses proliferation, triggers autophagy-mediated apoptotic cell death, and reduces metastatic potential in colon cancer cells. These findings strongly support the need for further evaluation of **3a**’s therapeutic potential through advanced mechanistic approaches and in vivo models.

## Figures and Tables

**Table 1:** In vitro antiproliferative activities of synthesized compounds (**3a**–**b**, **4a**–**b**) and cisplatin against breast (MDA-MB-231, 4T1) and colon (HT-29, DLD-1) cancer cell lines following 72 h of treatment. Data represent the mean IC_50_ values determined by the MTT assay.

IC_50_ (µM)				
	MDA-MB-231	4T1	HT-29	DLD-1
**3a**	8.2	6.4	3.7	7.2
**3b**	8.7	10.2	28.6	43.4
**4a**	100<	100<	100<	100<
**4b**	100<			
Cisplatin	10.3	4.6	3.1	8.5

**Table 2:** Docking results of compound **3a** with BAX, BCL2, and Caspase-3, showing binding energy (kcal/mol), RMSD (A°), and interacting residues.

Targets	Binding Energy	RMSD	Interacted Residues
BAX	−4.89 kcal/mol	0.14 A°	Ala24, Leu47, Val50, Arg134, Met137, Asp142, Arg145
BCL2	−6.06 kcal/mol	0.59 A°	Asp108, Phe109, Phe101, Met112, Ala146, Val153
Caspase 3	−6.29 kcal/mol	0.39 A°	Ser120, His121, Gln161, Cys163, Leu168, Tyr204, Trp206

## Data Availability

The data supporting the reported results of this study are available upon reasonable request from the corresponding author.
